# Obesity may not be related to pathologic response in locally advanced rectal cancer following neoadjuvant chemoradiotherapy

**DOI:** 10.3389/fonc.2022.994444

**Published:** 2022-09-29

**Authors:** Quoc Riccardo Bao, Filippo Crimì, Giovanni Valotto, Valentina Chiminazzo, Francesca Bergamo, Alessandra Anna Prete, Sara Galuppo, Badr El Khouzai, Emilio Quaia, Salvatore Pucciarelli, Emanuele Damiano Luca Urso

**Affiliations:** ^1^ General Surgery 3, Department of Surgical- Oncological and Gastroenterological Sciences DiSCOG, University of Padova, Padova, Italy; ^2^ Institute of Radiology - Department of Medicine, University of Padova, Padova, Italy; ^3^ Unit of Biostatistics, Epidemiology and Public Health, Department of Cardiac, Thoracic, Vascular Sciences and Public Health, University of Padova, Padova, Italy; ^4^ Unit of Medical Oncology 1, Veneto Institute of Oncology IOV - IRCCS, Padova, Italy; ^5^ Radiotherapy Unit, Veneto Institute of Oncology IOV - IRCCS, Padova, Italy

**Keywords:** radiologic fat parameters and rectal cancer outcomes rectal cancer, neoadjuvant chemoradiotherapy, pathological response, obesity, visceral fat, BMI

## Abstract

**Background:**

The aim of this study is to evaluate the correlation between body mass index (BMI) and body fat composition (measured with radiological fat parameters (RFP)) and pathological response after neoadjuvant chemoradiotherapy for locally advanced rectal cancer patients. The secondary aim of the study was to assess the role of BMI and RFP on major surgical complications, overall survival (OS), and disease-free survival (DFS).

**Methods:**

All patients who underwent surgical resection following nCRT between 2005 and 2017 for mid-low rectal cancer were retrospectively collected. Visceral fat area (VFA), superficial fat area (SFA), visceral/superficial fat area ratio (V/S), perinephric fat thickness (PNF), and waist circumference (WC) were estimated by baseline CT scan. Predictors of pathologic response and postoperative complications were investigated using logistic regression analysis. The correlations between BMI and radiologic fat parameters and survival were investigated using the Kaplan–Meier method and log-rank test.

**Results:**

Out of 144 patients included, a complete (TRG1) and major (TRG1+2) pathologic response was reported in 32 (22%) and 60 (45.5%) cases, respectively. A statistically significant correlation between BMI and all the RFP was found. At a median follow-up of 60 (35–103) months, no differences in terms of OS and DFS were found considering BMI and radiologic fat parameters. At univariable analysis, neither BMI nor radiologic fat parameters were predictors of complete or major pathologic response; nevertheless, VFA, V/S>1, and BMI were predictors of postoperative major complications.

**Conclusions:**

We found no associations between BMI and body fat composition and pathological response to nCRT, although VFA, V/S, and BMI were predictors of major complications. BMI and RFP are not related to worse long-term OS and DFS.

## Introduction

Rectal cancer represents a major cause of morbidity and mortality worldwide, and the actual standard of care for locally advanced rectal cancer is total mesorectal excision (TME) following neoadjuvant chemoradiotherapy (nCRT) (1).

A high body mass index (BMI) was associated with an increased colorectal cancer risk (2, 3), and general and visceral obesity were reported as risk factors for the increased incidence of colorectal neoplasms (4). Furthermore, BMI has been linked to a worse outcome of colorectal cancer (5–8), probably due to the deregulation of IGFR-1 and other cytokines involved in metabolic syndrome, which are overexpressed in obese patients (9). IGFR-1 was correlated with a poor response after nCRT in rectal cancer (10), and visceral obesity was associated with worse outcomes in patients with stage II and III colorectal cancer in terms of surgical outcomes and recurrence (11–14).

The impact of obesity and visceral fat on response to neoadjuvant treatment was investigated in other neoplasms, such as breast cancer. Even with controversial results, the role of visceral fat in the mechanism of chemosensitivity was suggested (15, 16). In rectal cancer, obese patients were reported to have a lower rate of complete pathologic response (pCR) and a lower rate of sphincter-preserving procedures. However, no difference in terms of recurrence rate was described in obese and nonobese (6). Furthermore, Sun et al. confirmed that obese patients have a lower pCR rate, besides BMI was associated with an adverse effect on downstaging and tumor regression grade, and resulted as a strong predictor for recurrence (5).

Up to 20% of rectal cancer patients showed a pCR following nCRT, permitting also organ-sparing approaches. The identification of predictors of pathologic response, such as biological markers (i.e., carcinoembryonic antigen (CEA), microsatellite instability) (17, 18), is now essential to the best selection of patients in a rectum-sparing program. However, the impact of obesity and body fat composition on pathologic response has not been extensively assessed in the current literature.

The aim of this study is to evaluate the correlation between obesity (defined using BMI) and body fat composition (measured with radiological fat parameters (RFP)) and pathological response after nCRT for locally advanced rectal cancer patients. The secondary aim of the study was to assess the role of BMI and RFP on major surgical complications, overall survival (OS), and disease-free survival (DFS).

## Methods

### Patients’ selection

All patients who underwent surgical resection following nCRT for locally advanced rectal cancer between 2005 and 2017 were retrospectively collected from the prospectively maintained database of the Colorectal Surgery (General Surgery 3), University Hospital of Padova. The study was notified and approved by the local Ethical Committee. Inclusion criteria were histologically confirmed mid-low rectal adenocarcinoma up to 12 cm from the anal verge surgically treated following standard nCRT. For patients treated with upfront or emergency surgery, or with recurrent disease, short-course radiotherapy was excluded. The baseline work-up included clinical history, digital rectal examination, colonoscopy, CEA level, chest/abdomen computed tomography (CT) scan, and pelvic magnetic resonance imaging (MRI).

Clinical and pathological TNM staging were reported according to the American Joint Committee on Cancer (AJCC) Eighth Edition (19). Tumor regression grade (TRG) was assessed according to Mandard’s classification (20). pCR was defined as no viable tumor cell found in the surgical specimen (TRG1), while major pathologic responses as TRG1 and TRG2.

### Treatment details

All the patients underwent standard nCRT with 5-FU/capecitabine and 50.4 Gy of fractioned radiotherapy. Patients who underwent short-course radiotherapy (5 × 5) were excluded to eliminate a possible confounding factor. Indication for nCRT was discussed during the multidisciplinary meeting, according to current guidelines (21, 22). Surgical resection was planned after a re-evaluation during the multidisciplinary meeting. If a complete or major clinical response was observed (23) and patients were eligible for a rectum-sparing approach in the context of other study protocols, currently running in our center (23–25), patients were treated with local excision [i.e., transanal excision, transanal endoscopic operation (TEO)]. On the contrary, if a partial/absent response was observed, or in the case of patients not eligible for rectum-sparing approach, a radical resection (i.e., TME), as low anterior resection (LAR), abdominoperineal resection (APR), or intersphinteric resection (ISR) with coloanal anastomosis, was planned. In patients treated by local excision, radicalization surgery (completion TME) was recommended when one of the high-risk features was present on the histopathological report as previously described (25). Patients treated with a rectum-sparing approach were followed up every 3 months according to study protocols (23–25). In patients treated with TME, adjuvant treatment was offered to patients with pTNM II stage with high-risk features or pTNM III stage according to national guidelines (22).

### Obesity indexes and radiological fat parameters

**Figure 1:**
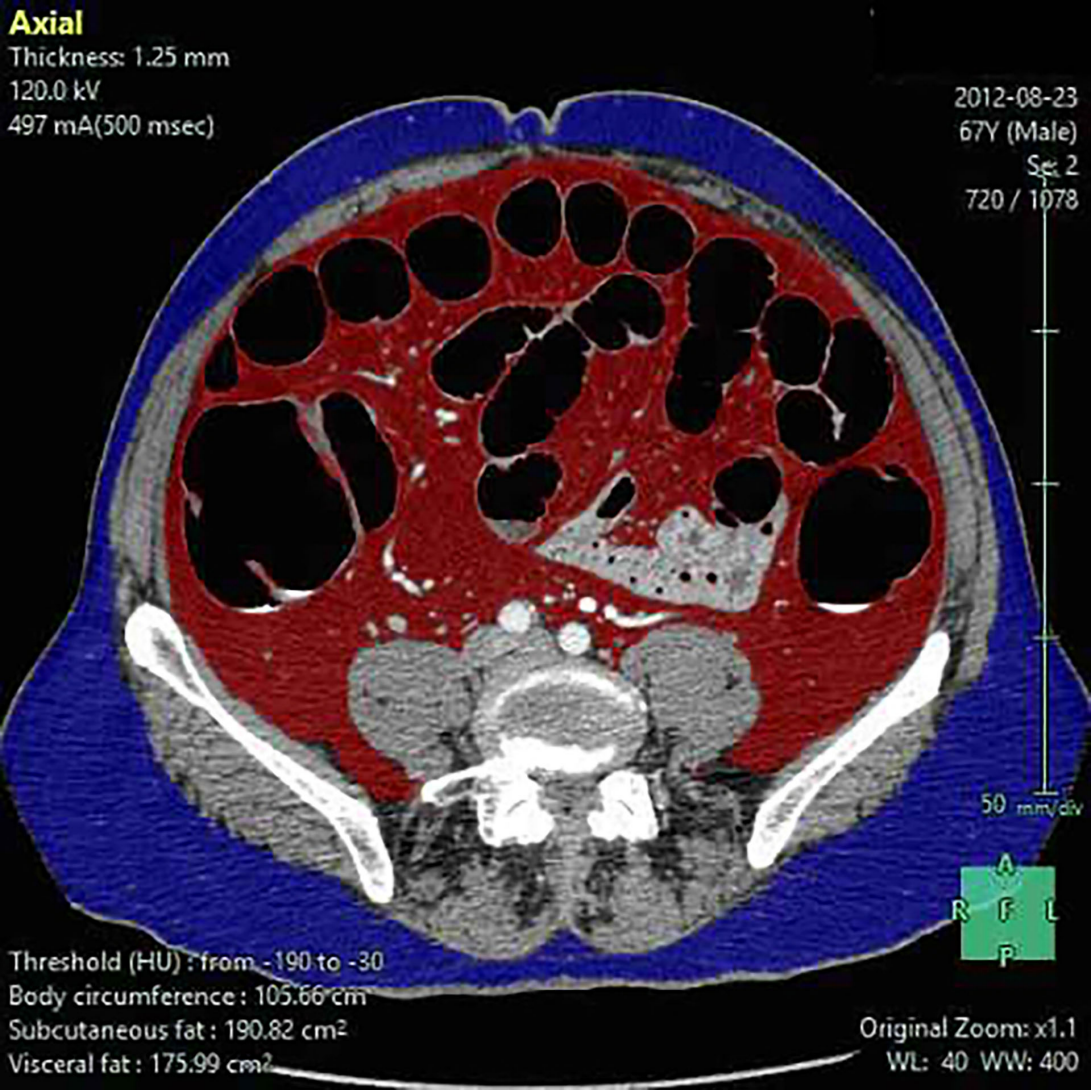
Visceral (VFA), superficial fat area (SFA), and waist circumference (WC) estimate. Superficial fat (blue), visceral fat (red), and waist circumference are estimated by using reconstruction software (Fujifilm Synapse 5).

BMI was calculated at baseline assessment as the ratio between body weight (kg)/height (m^2^). For each patient, abdominal fat was calculated as previously described from the available preoperative baseline CT scan by an expert radiologist, who was blinded to clinical data (26) using reconstruction software (Fujifilm Synapse 5). The following RFP were estimated: superficial fat area (SFA), visceral fat area (VFA), total fat area (TFA), perinephric fat (PNF), waist circumference (WC), and visceral/superficial fat area ratio (V/S). The image attenuation range was set between −190 and −30 Hounsfield Unit (HU) (11). VFA and SFA were measured using a single slice at the level of the intervertebral space between L4 and L5 (**Figure 1**). The area of the psoas and sacrospinal muscles were excluded from the area since it may contain fatty tissue derived from age-related fatty degeneration (27). TFA was calculated by summing VFA and SFA. PNF was defined as the shortest distance (mm) between the kidney and the abdominal wall (28). WC was calculated at the level of the middle point between the last rib and the iliac crest (29). V/S was calculated as the ratio between VFA and SFA.

### Statistical analysis

Continuous variables were reported as median (I–III quartiles), while qualitative variables were reported as absolute numbers and percentages. Descriptive statistical analysis was performed by dividing patients with pCR and non-pCR, and complete/major vs partial/absent pathological response (TRG3–5). Significant differences between the two groups were tested by Pearson’s Chi-square for categorical variables and the Mann–Whitney *U* test for continuous variables. PNF and BMI were evaluated as both continuous and categorical variables, using as cutoff the median value (14.7 mm) for PNF and generally accepted cutoffs defined for overweight (BMI>25) and obesity (BMI>30). For V/S, two cutoffs of 0.4 and 1 were used according to the cutoffs used in the previous literature (11, 28). The correlation between BMI and RFP was evaluated with Spearman’s correlation coefficient and graphically presented using a correlation plot. Predictors of pathologic response were investigated using a univariable logistic regression approach, and a multivariable model was planned to investigate the independent predictors. The Kaplan–Meier method was used to estimate the OS, DFS, local-recurrence free survival (LRFS), and distant-recurrence free survival (DRFS). The survival curves were compared using the log-rank test. Local recurrence (LR) was defined as any recurrence in the pelvis, while distant recurrence (DR) was defined as any other recurrence. The association between RFP and OS and DFS was evaluated with univariable Cox proportional hazard models. All statistical analyses were performed using R software (version 4.0.3) (30), using the RMS package (31).

## Results

### Patients, tumor, and treatment characteristics

Patients, tumor, and treatment characteristics are summarized in **Table 1**. Overall, 144 patients were included for analysis, 97 (67.4%) were men and 47 (32.6%) were women. The median age was 66 (58–74) years, the median distance of the tumor from the anal verge was 6.0 (4.0–9.0) cm, and the median preoperative CEA was 2.1 (1.2–4.2) ng/ml.

**Table 1:** Patients, tumor, and treatment characteristics.

	BMI < 25		BMI > 25		BMI < 30		BMI > 30		Total	
	*N* = 70	% or IQR	*N* = 74	% or IQR	*N* = 128	% or IQR	*N* = 16	% or IQR	*N* = 144	% or IQR
**Sex**										
Male	40	57	57	77	87	68	10	62	97	67.4
Female	30	43	17	23	41	32	6	38	47	32.6
Age										
Median (years)	63	55–72	68	60–77	65	56–73	70	66–76	66	58–66
**BMI**										
Median	22.6	21.2–23.9	26.9	26.2–29.5	24.5	22.5–26.5	32.7	31.1–36.6	25.0	22.7–27.1
**PNF**										
<14.7	47	71	21	30	63	52	5	31	68	49.7
≥14.7	19	29	50	70	58	48	11	69	69	50.3
**V/S**										
<0.4	12	16	5	4	15	10	2	12	17	12.0
≥0.4	58	84	69	96	113	90	14	88	124	88.0
<1	50	71	43	60	79	63	14	88	93	66.0
≥1	19	28	29	40	46	37	2	12	48	34.0
**Clinical T stage**										
cT2	14	20	6	8	19	15	1	6	20	13.9
cT3	37	53	46	62	71	55	12	75	83	57.6
cT4	19	27	22	30	38	30	3	19	41	28.5
**Clinical N stage**										
cN0	7	10	8	11	13	10	2	12	15	10.4
cN+	63	90	66	89	115	90	14	88	129	89.6
**Surgical procedure**										
Low anterior resection	44	63	47	64	82	64	9	56	91	63.2
Abdominoperineal resection	11	16	13	18	20	16	4	25	24	16.7
Local excision	10	14	10	14	17	13	3	19	20	13.9
Intersphinteric resection	5	7	4	4	9	7	0	0	9	6.3
**Re-operation**										
No	67	96	66	89	120	94	13	81	133	92.4
Yes	3	4	8	11	8	6	3	19	11	7.6
**Grading**										
G*X*	6	10	8	13	12	11	2	20	14	11.2
G1	8	13	10	16	18	16	0	0	18	14.4
G2	34	56	33	52	60	53	7	70	67	54.4
G3	13	21	12	19	24	21	1	10	25	20.0
**Pathological T stage**										
ypT0	18	26	14	19	27	21	5	31	32	22.2
ypTis	3	4	1	1	3	2	1	6	4	2.8
ypT1	8	11	7	9	15	12	0	0	15	10.4
ypT2	10	14	27	36	33	26	4	25	37	25.7
ypT3	26	37	23	31	43	34	6	38	49	34.0
ypT4	5	7	2	3	7	5	0	0	7	4.9
**Pathological N stage**										
ypN*X*	11	16	9	12	17	13	7	44	20	13.9
ypN0	40	57	44	59	77	60	7	44	84	58.3
ypN1	11	16	16	22	22	17	5	31	27	18.7
ypN2	8	11	5	7	12	9	1	6	13	9.0
**Tumor regression grade**										
TRG1	20	29	15	22	29	24	6	40	35	24.2
TRG2	12	18	16	24	26	22	2	13	28	21.2
TRG3	16	24	24	36	37	31	3	20	40	30.3
TRG4	16	24	10	15	23	19	3	20	26	19.7
TRG5	4	6	2	3	5	4	1	7	6	4.5

BMI, body mass index; PNF, perinephric fat; V/S, visceral/superficial fat area ratio; TRG, tumor regression grade.

The median time from the completion of nCRT and surgery was 8.6 (7.0–11.4) weeks. After nCRT, 91 (63.2%) patients underwent LAR, 24 (16.7%) APR, 20 (13.9%) local excision, and nine (6.3%) ISR with coloanal anastomosis. Among TME procedures, 106 (73.6%) patients had an open traditional approach and 18 (12.5%) had a laparoscopic approach. Among the 20 patients treated with local excision, only three (15%) had negative histopathological features and required a completion TME according to the study protocol. BMI, RFP, and postoperative complications according to Clavien–Dindo are described in **Supplementary Table S2**. Postoperative complications occurred in 55 (38.2%) patients, and in 11 (7.6%) patients requiring re-operation (Clavien–Dindo >3a). The histopathological analysis reported a pCR in 32 (22.2%) patients, whereas a major pathologic response in 28 (21.2%), respectively.

### Obesity and radiological fat parameters

**Figure 2:**
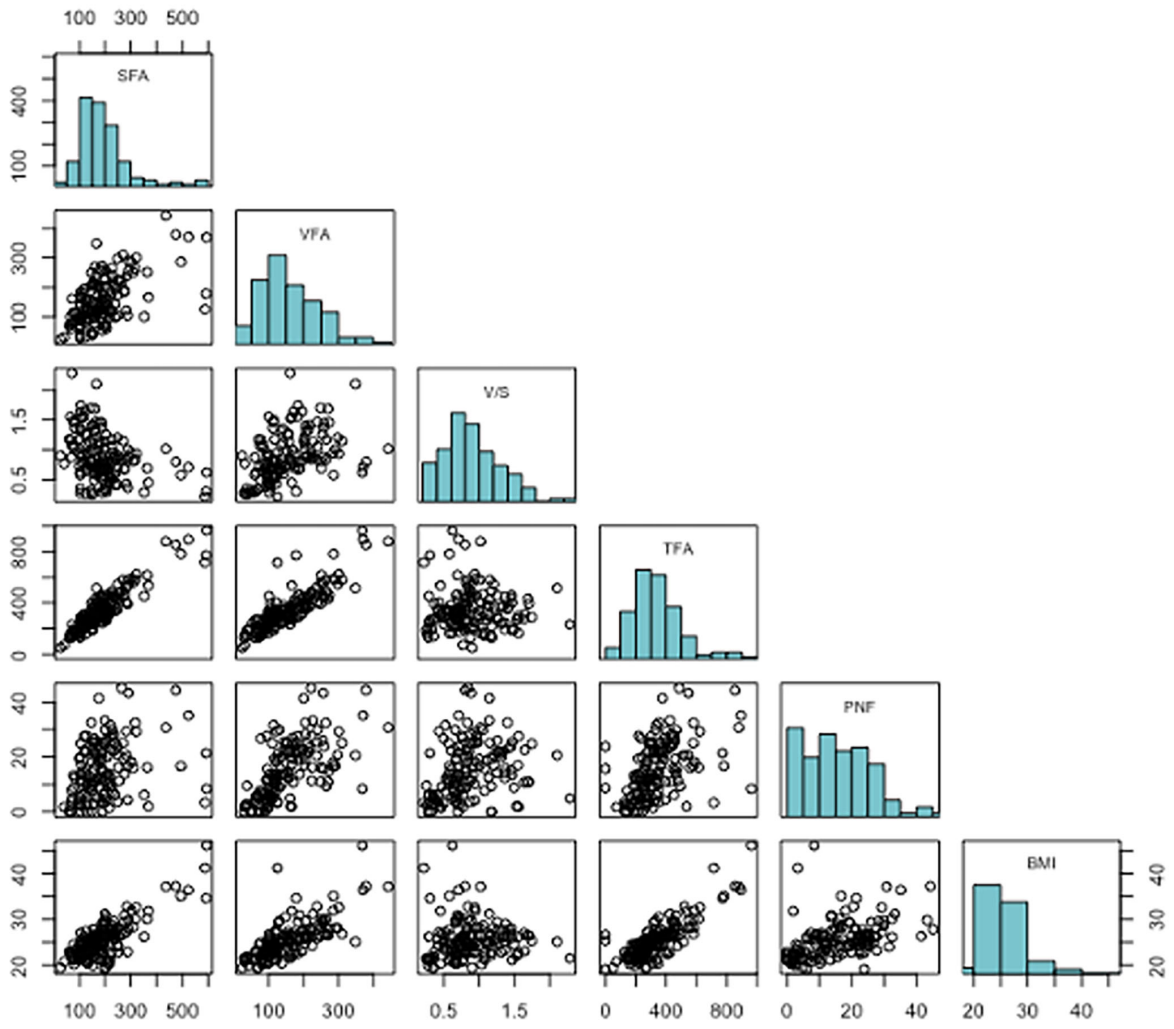
Correlation plot among BMI and radiological fat parameters.

Median BMI was 25.0 (22.7–27.0), median SFA, VFA, and TFA were 175.5 (124.8–227.6), 140.8 (99.9–205.1), and 318 (249–430) cm^2^, respectively. Median PNF was 14.7 (7.4–22.6) mm, and median WC was 95.7 (88.4–103.8) cm. Median V/S ratio was 0.827 (0.620–1.141). The Spearman’s correlation coefficient showed a statistically significant correlation between SFA (*p* = 0.63, *p* < 0.001), VFA (*p* = 0.76, *p* < 0.001), V/S (*p* = 0.17, *p* = 0.04), TFA (*p* = 0.78, *p* < 0.001) PNF (*p* = 0.55, *p* < 0.001), and BMI (**Figure 2**).

### Complete and major pathologic response

The clinicopathological characteristics of patients with a pCR and a complete/major pathological response are summarized in **Table 2** and **Supplementary Table S1**. A statistically significant difference was found between pCR and non-pCR patients regarding preoperative CEA (*p* = 0.04), distance from the anal verge (*p* = 0.002), and baseline cT stage (*p* = 0.03). No difference between the group pCR or a major pathological response regarding BMI, SFA, VFA, TFA, PNF, WC, or V/S.

**Table 2:** Clinicopathological characteristics of patients with a ypT0 vs. ypT1/2/3/4.

	ypT0 (*n* = 32)	ypT1/2/3/4 (*n* = 112)	Total (*n* = 144)	*p*-values
**Sex**				
Female (%)	13 (41)	34 (30)	47 (33)	0.275
Male (%)	19 (59)	78 (70)	97 (67)	
**BMI**				
Median (Iqr)	24.2 (22.4–26.7)	25.1 (22.9–27.1)	25.0 (22.7–27.1)	0.514
**SFA**				
Median (Iqr)	195.2 (134.9–221.5)	167.5 (124.8–228.3)	175.5 (124.8–227.6)	0.518
**VFA**				
Median (Iqr)	132.6 (93.1–177.5)	143.1 (101.1–211.2)	140.8 (99.9–205.1)	0.324
**TFA**				
Median (Iqr)	336 (248–397)	313 (249–440)	318 (249–430)	0.743
**PNF**				
Median (Iqr)	12.3 (3.8–20.9)	15.7 (8.5–23.0)	14.7 (7.4–22.6)	0.211
**WC**				
Median (Iqr)	95.3 (86.4–102.0)	95.9 (88.8–104.5)	95.7 (88.4–103.8)	0.232
**V/S**				
Median (Iqr)	0.8 (0.5–1.0)	0.8 (0.7–1.2)	0.8 (0.6–1.1)	0.074
**V/S**				
<0.4	7 (22)	10 (9)	17 (12)	0.052
≥04	25 (78)	99 (91)	124 (88)	
**V/S**				
<1	25 (78)	68 (62)	93 (66)	0.098
≥1	7 (22)	41 (38)	48 (34)	
**CEA**				
Median (Iqr)	1.5 (1.1–2.8)	2.3 (1.3–4.8)	2.1 (1.2–4.2)	**0.042**
**Distance a.v.**				
Median (Iqr)	7.5 (6.0–9.3)	5.0 (3.8–8.0)	6.0 (4.0–9.0)	**0.002**
**Distance a.v.**				
<5 cm	4 (12)	41 (37)	45 (31)	**0.007**
≥5 cm	28 (88)	71 (63)	99 (69)	
**cT stage**				
2	6 (19)	14 (12)	20 (14)	**0.025**
3	23 (72)	60 (54)	83 (58)	
4	3 (9)	38 (34)	41 (28)	
**cN stage**				
0	2 (6)	13 (12)	15 (10)	0.382
1	30 (94)	99 (88)	129 (90)	
**Grading**				
G1	7 (29)	11 (11)	18 (14)	**< 0.001**
G2	8 (33)	60 (59)	68 (54)	
G3	2 (8)	23 (23)	25 (20)	
G*X*	7 (29)	7 (7)	14 (11)	
**pN stage**				
N0	18 (56)	66 (59)	84 (58)	**0.001**
N1	2 (6)	25 (22)	27 (19)	
N2	0 (0)	13 (12)	13 (9)	
N*X*	12 (38)	8 (7)	20 (14)	
PNF				
<14.7	18 (58)	51 (48)	69 (50)	0.33
≥14.7	13 (42)	55 (52)	68 (50)	
**BMI**				
<25	18 (56)	55 (49)	73 (51)	0.476
≥25	14 (44)	57 (51)	71 (49)	
**BMI**				
<30	27 (84)	101 (90)	128 (89)	0.357
≥30	5 (16)	11 (10)	16 (11)	

BMI, body mass index; SFA, superficial fat area; VFA, visceral fat area; TFA, total fat area; V/S, visceral/superficial fat area ratio; PNF, perinephric fat; WC, waist circumference; TRG, tumor regression grade. Bold values are statistically significant values.

### Long-term outcomes and prognostic factors

Following a median follow-up of 59 (20–104) months, 20 (14.1%) patients died and 36 (25.0%) experienced recurrence. Five (3.4%) of these patients had LR, 27 (18.8%) had DR, and four (2.8%) had both LR and DR. The median OS of the whole cohort was 60.0 (34–104) months, and the median DFS was 32.0 (12.2–66.0) months. In patients with LR, the median LRFS was 18 (17–20) months, whereas the median DRFS was 15 (9–25) months. Out of the 20 patients treated with local excision, the median follow-up was 65.5 (48.8–110) months. Of these, one patient suffered LR requiring salvage TME, one patient had DR, and one patient had both LR and DR.

**Figure 3:**
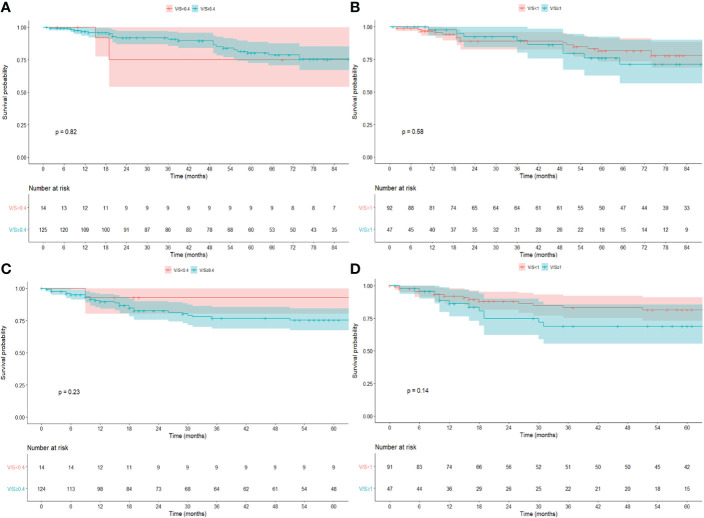
Kaplan–Meier survival estimate for OS and DFS for V/S using a cutoff of 0.4 **(A, C)** and 1.0 **(B, D)**.

No differences in terms of OS, DFS, LRFS, and DRFS were found considering PNF (log-rank *p* = 0.89, *p* = 0.63, *p* = 0.38, and *p* = 0.72, respectively) (**Supplementary Figure S1**), BMI>25 (log-rank *p* = 0.66, *p* = 0.46, *p* = 0.48, and *p* = 0.51, respectively), BMI>30 (log-rank *p* = 0.55, 0.82, *p* = 0.93, and *p* = 0.99, respectively) (**Supplementary Figure S2**), V/S using a cutoff of 0.4 (log-rank *p* = 0.82, *p* = 0.23, *p* = 0.85, and *p* = 0.24, respectively), and a cutoff of 1 (log-rank *p* = 0.58, *p* = 0.14, *p* = 0.30, and *p* = 0.19, respectively) (**Figure 3**).

### Logistic regression analysis

In a univariable logistic regression (**Table 3**) analysis, the baseline cT stage (OR, 4.86 (95% CI, 1.36–17.29); *p* = 0.04) and distance from the anal verge (OR, 0.22 (95% CI, 0.16–0.56); *p* = 0.00) were found to be predictors of pCR. Sex (OR, 0.42 (95% CI, 0.20–0.85); *p* = 0.02) and preoperative CEA (OR, 0.275 (95% CI, 1.07–1.98); *p* = 0.01) were linked to a significant pathological response. The general obesity index (BMI, WC) and abdominal obesity (SFA, VFA, TFA, and V/S ratio) were not predictive of pCR or major pathological response (TRG1–2).

**Table 3:** Logistic regression univariable analysis.

	Pathologic complete response			Pathologic major response		
	OR	95% CI	*p*-value	OR	95% CI	*p*-value
**Sex**	1.566	0.70–3.54	0.28	0.418	0.20–0.85	**0.02**
**Age**	1.409	0.82–2.43	0.22	1.374	0.85–2.23	0.2
**BMI**	1.101	0.72–1.70	0.63	0.867	0.61–1.23	0.43
**SFA**	0.974	0.66–1.44	0.9	1.020	0.73–1.42	0.91
**VFA**	1.424	0.81–2.50	0.22	0.892	0.57–1.38	0.61
**TFA**	1.13	0.72–1.78	0.6	0.967	0.67–1.40	0.86
**PNF**	1.555	0.83–2.92	0.17	0.680	0.41–1.14	0.14
**V/S**	0.582	0.33–1.04	0.07	0.765	0.49–1.20	0.24
**V/S < 0.4 vs. ≥ 0.4**	0.361	0.12–1.04	0.06	1.978	0.71–5.54	0.19
**V/S < 1 vs. ≥ 1**	0.464	0.18–1.17	0.1	1.497	0.73–3.05	0.27
**CEA < 5 vs. ≥ 5**	0.497	0.16–1.55	0.23	0.275	1.07–1.98	**0.01**
**Distance AV < 5 vs. ≥ 5**	0.215	0.08–0.56	**< 0.01**	1.909	0.97–3.76	0.06
**cT stage**	4.855	1.36–17.29	**0.04**	0.717	0.33–1.55	0.56
**cN stage**	1.970	0.42–9.22	0.39	2.151	0.72–6.40	0.17
**PNF < 14.7 vs. ≥ 14.7**	1.493	0.66–3.35	0.33	0.637	0.32–1.26	0.19
**BMI < 25 vs. ≥ 25**	1.332	0.60–2.94	0.48	0.837	0.43–1.62	0.6
**BMI < 30 vs. ≥ 30**	0.588	0.19–1.84	0.36	1.370	0.48–3.88	0.55

BMI, body mass index; SFA, superficial fat area; VFA, visceral fat area; TFA, total fat area; V/S, visceral/superficial fat area ratio; PNF, perinephric fat; WC, waist circumference. Bold values are statistically significant values.

### Predictors of postoperative complications

At logistic regression analysis, VFA (OR, 2.14 (95% CI, 1.05–4.38)), V/S>1 (OR, 0.04 (95% CI, 0.04)), and BMI (OR, 1.71 (95% CI, 1.04–2.82)) were predictors of postoperative major complications.

## Discussion

The present study failed to demonstrate the correlation of BMI and RFP to pathologic response after nCRT and to the long-term outcomes in locally advanced rectal cancer patients. In our findings, general obesity and visceral fat did not correlate with pathologic response, so these parameters are not to be considered contraindications for the organ-sparing approach. Moreover, obesity and visceral fat were confirmed to be predictors of postoperative major complications. Obesity is known to be associated with increased intra- and postoperative complications, and some studies reported a worse survival after surgery in rectal cancer patients after nCRT (32–34). The role of obesity and abdominal fat as prognostic factors and their impact on oncological short- and long-term outcomes were studied with controversial results. We found no significant association between obesity and radiological abdominal fat parameters considered (BMI, SFA, VFA, TFA, PNF, WC, and V/S) and the pathologic response to nCRT. In the previous literature, a few authors investigated the correlation between obesity or abdominal fat and oncological outcomes in rectal cancer after nCRT (5, 6, 28, 35), whereas others investigated the role of obesity indexes in colon and rectal cancer patients altogether (14, 33). Park et al. and Sun et al. reported on two large series of rectal cancer patients and correlated only BMI to oncological outcomes (5, 6). Park et al. reported a lower rate of pCR and a lower rate of sphincter-saving procedures in obese patients (patients with a BMI>30) (6). Similarly, Sun et al. reported that obese patients had a lower pCR rate and adverse effects on downstaging and TRG. In this study, BMI>30 was found to be a strong predictor of recurrence, with an increased 5-year LR rate in severely obese patients (5). On the other hand, in both studies, OS was not affected by BMI. However, these authors used only BMI as an obesity index, instead of a more specific radiological index such as VFA and V/S.

Han et al. (36) described the association between obesity (defined as a BMI >25) and visceral obesity (defined as a VFA ≥100) and pCR in 536 rectal cancer patients after nCRT without finding any statistical correlation between those parameters. Similarly to our study, Lee et al. investigated the role of RFP in 125 rectal cancer patients. They found that only V/S>1 was related to a higher recurrence rate, and a worse DFS and OS. However, this study did not include patients treated with nCRT (11). AV/S cutoff of 0.4 was used by Clark et al., which found that higher VFA, V/S, and BMI were related to a minor tumor downstaging, a decreased DFS, and an increased recurrence rate. Furthermore, PNF was associated with a worse OS (28). Interestingly, patients with a V/S>0.4 were statistically older and affected by other comorbidities (hypertension, hypercholesterolemia). These conditions could explain a trend toward a worse OS. Moon et al. demonstrated that a higher V/S was related to a lower DFS, without difference in terms of OS (14). Finally, Goulart et al., including colon and rectal cancer patients, reported no difference in OS and DFS by dividing VFA into quartiles (33).

In our study, BMI and VFA were confirmed to be associated with postoperative complications. It is widely assumed that surgery in obese patients is affected by increased postoperative comorbidity due to the more difficult surgery and all the comorbidity associated with metabolic syndrome. Similarly to our finding, Zhou et al. reported VFA as a strong independent predictor of postoperative complications in rectal cancer (32). However, in this study, all the patients treated with nCRT were excluded. Heus et al. reported VFA and TFA in rectal cancer patients undergoing long-course nCRT. Using a cutoff of 100 cm^2^ for VFA, an increase in operative blood loss and postoperative complications were reported (35). Even if the role of postoperative complications on the long-term oncological outcome is still debated (37), postoperative complications, as far as worse general performance status, may result in a delay in adjuvant therapy and in an increased rate of LR and a decreased survival (38, 39).

The available literature on rectal cancer patients, obesity parameters, pCR, and other oncological outcomes has given conflicting results, mainly because in different studies, there are different inclusion criteria, methods, and main objectives and endpoints. With these limitations, there is no agreement on the role of obesity and its related radiological parameters on oncological outcomes in locally advanced rectal cancer patients treated with nCRT and surgery. Conflicting data exist on the role of obesity index on pathological response to nCRT, and only BMI was considered by Sun et al. and Park et al. (the studies with the largest number of patients included), while RFPs were not investigated (5, 6). Unlike oncological outcomes, we can find agreement in the literature on finding an association between most obesity index and perioperative complications. Based on the data available in the present study, obesity cannot basically influence oncological decision-making, but it is a predictor of a higher rate of surgical complications.

Our study does have some limitations. First is the small number of enrolled patients when compared with a few similar previous publications, even if only the study of Clark et al. has the same inclusion criteria and the same methods we used (28). Clark et al. analyzed the same obesity parameters we considered in a group of 99 rectal cancer patients treated with nCRT, finding that elevated V/S or PNF was associated with shorter DFS and OS. The relationships between BMI, RFPs, pathologic response, and postoperative complications were not investigated. From this point of view, our study investigated the largest group of locally advanced rectal cancer patients, all surgically treated after a neoadjuvant approach, considering BMI and all the most known RFPs, in association with pCR, OS, DFS, and perioperative complications. Despite these considerations, the relatively small number of patients enrolled could cover the predictive prognostic potential of some of the parameters studied in many of the analyses presented. For this reason, also, we did not use a multivariable model to investigate the independent predictors of complete/major pathologic response since none of the parameters considered resulted as a predictor of pathologic response.

Second, its retrospective design, even if the clinical data were prospectively maintained in our database, whereas RFP was retrospectively collected from CT scans, and the number of enrolled patients is smaller than in other studies. Third, we arbitrarily used different cutoffs for the RFP considered since there is no strong evidence, nor agreement, in the current literature about this topic. Further studies are needed to establish the proper cutoff of these indexes. Furthermore, we considered altogether different surgical procedures such as TME and local excision. Considering that the primary aim of the study is to assess a correlation between body fat and tumor regression, we think that patients enrolled in an organ-preservation prospective clinical study (ReSARCH trial) (23) with an accurate and standardized histopathological analysis and a long-term follow-up are eligible for the analysis, even if no pathological data are available on their mesorectal status. Last, we are analyzing the effect of nCRT on an Italian cohort, where the median BMI ranges between 24 and 26, and the rate of obesity and overweight is 10% and 35%, respectively (40). To note, this is the first study to analyzed the relationship between obesity and oncological outcomes in a group of Italian rectal cancer patients, whereas most of the cited studies are from the USA or China, where the estimated rate of obesity is greater than 36% and 16%, respectively (41, 42).

## Conclusions

We found no associations between BMI and RFP and pathological response to nCRT, although VFA, V/S, and BMI were predictors of major surgical complications. BMI and RFP are not related to worse long-term OS and DFS.

## Data availability statement

The raw data supporting the conclusions of this article will be made available by the authors, without undue reservation.

## Ethics statement

The studies involving human participants were reviewed and approved by Comitato Etico per la Sperimentazione Clinica della Provincia di Padova. Written informed consent for participation was not required for this study in accordance with the national legislation and the institutional requirements.

## Author contributions

Study concepts: QB, FC, EQ, SP, EU. Study design: QB, FC, EU. Data acquisition: QB, FC, GV, FB, AP, SG, BK. Data analysis and interpretation: QB, FC, GV, FB, AP, SG, BK. Statistical analysis: FC, VC. Manuscript preparation: QB, FC, GV. Manuscript editing: VC, FB, AP, SG, BK. Manuscript review: QB, EQ, SP, EU. All the authors revised and approved the final version of the manuscript.

## Conflict of interest

The authors declare that the research was conducted in the absence of any commercial or financial relationships that could be construed as a potential conflict of interest.

## Publisher’s note

All claims expressed in this article are solely those of the authors and do not necessarily represent those of their affiliated organizations, or those of the publisher, the editors and the reviewers. Any product that may be evaluated in this article, or claim that may be made by its manufacturer, is not guaranteed or endorsed by the publisher.
