# Supplementary material for: Obesity may not be related to pathologic response in locally advanced rectal cancer following neoadjuvant chemoradiotherapy
Source: Front Oncol. 2022 Sep 29;12:994444. doi: 10.3389/fonc.2022.994444 (PMC9556820; doi:10.3389/fonc.2022.994444)

Supplementary file.

Table 1. Descriptive analysis non-pathological response (TRG3-5) Vs major pathological response (TRG1-2)

		**TRG 3/4/5** (n=82)	**TRG 1-2** (n=62)	**Total** (n=144)	**p-values**
**Sex**	Female (%)	20 (24)	27 (44)	47 (33)	**0.015**
	Male (%)	62 (76)	35 (56)	97 (67)	
**BMI**	Median (Iqr)	25.2 (22.8 - 27.6)	24.8 (22.5- 26.8)	25.0 (22.7 - 27.1)	0.495
**SFA**	Median (Iqr)	160.2 (120.9 - 229.1)	191.4 (131.0 - 226.4)	175.5 (124.8 - 227.6)	0.301
**VFA**	Median (Iqr)	143.1 (100.2 - 206.1)	132.6 (100.1- 188.0)	140.8 (99.9 - 205.1)	0.605
**TFA**	Median (Iqr)	311 (237 – 439)	336 (260 – 404)	318 (249 – 430)	0.795
**PNF**	Median (Iqr)	15.7 (8.7 - 23.2)	13.7 (6.2 - 22.2)	14.7 (7.4 - 22.6)	0.254
**WC**	Median (Iqr)	96.0 (90.4 -105.3)	95.4 (86.9 - 103.6)	95.7 (88.4 - 103.8)	0.236
**V/S**	Median (Iqr)	0.9 (0.7 - 1.2)	0.789 (0.6 - 1.1)	0.8 (0.6 - 1.1)	0.177
**V/S**	< 0,4	7 (9)	10 (16)	17 (12)	0.188
	≥ 0,4	72 (91)	52 (84)	124 (88)	
**V/S**	< 1	49 (62)	44 (71)	93 (66)	0.266
	≥ 1	30 (38)	18 (29)	48 (34)	
**CEA**	Median (Iqr)	2.4 (1.3 - 5.8)	1.6 (1.0 - 2.7)	2.1 (1.2 - 4.2)	**0.003**
**CEA**	< 5	56 (90)	59 (72)	115 (80)	**0.006**
	≥ 5	10 (6)	23 (28)	29 (20)	
**Distance a.v.**	Median (Iqr)	5.0 (4.0 - 8.8)	6.5 (4.0 - 9.0)	6.0 (4.0 - 9.0)	0.114
**Distance a.v.**	< 5cm	22 (35)	42 (51)	64 (44)	0.06
	≥ 5cm	65 (40)	40 (49)	80 (56)	
**cT stage**	2	10 (12)	10 (16)	20 (14)	0.557
	3	46 (56)	37 (60)	83 (58)	
	4	26 (32)	15 (24)	41 (28)	
**cN stage**	0	6 (7)	9 (15)	15 (10)	0.161
	1	76 (93)	53 (85)	129 (90)	
**Grading**	G1	7 (9)	11 (23)	18 (14)	**<0.001**
	G2	50 (64)	18 (38)	68 (54)	
	G3	19 (24)	6 (13)	25 (20)	
	GX	2 (3)	12 (26)	14 (11)	
**pN stage**	N0	45 (55)	39 (63)	84 (58)	**<0.001**
	N1	23 (28)	4 (6)	27 (19)	
	N2	12 (15)	1 (2)	13 (9)	
	NX	2 (2)	18 (29)	20 (13.9)	
**PNF**	< 14,7	35 (45)	34 (57)	69 (50)	0.193

	≥ 14,7	42 (55)	26 (43)	68 (50)	
**BMI**	< 25	40 (49)	33 (53)	73 (51)	0.597
	≥ 25	42 (51)	29 (47)	71 (49)	
**BMI**	<30	74 (90)	54 (87)	128 (89)	0.552
	≥ 30	8 (10)	8 (13)	16 (11)	

Table 2. BMI, and radiologic fat parameters and post-operative complications according to Clavien Dindo score.

	Clavien Dindo <3a	Clavien Dindo>3a	Total
	**N=44(%)**	**N=11(%)**	**N=55**
**BMI>25**	21 (72.4)	8 (27.6)	29
**BMI>30**	8 (72.7)	3 (27.3)	11
**SFA>175.5)**	19 (76.0)	6 (24.0)	25
**VFA>140.8**	21 (72.4)	8 (27.6)	29
**TFA>318**	18 (72.0)	7 (28)	25
**PNF>14.7**	15 (68.2)	7 (31.8)	22
**V/S>0.4**	35 (77.7)	10 (22.2)	45
**V/S>1**	12 (63.2)	7 (36.8)	19

Figure 1. Kaplan-Meier survival estimate for OS (a) and DFS (b) for PNF. PNF cut-off=14.7mm.

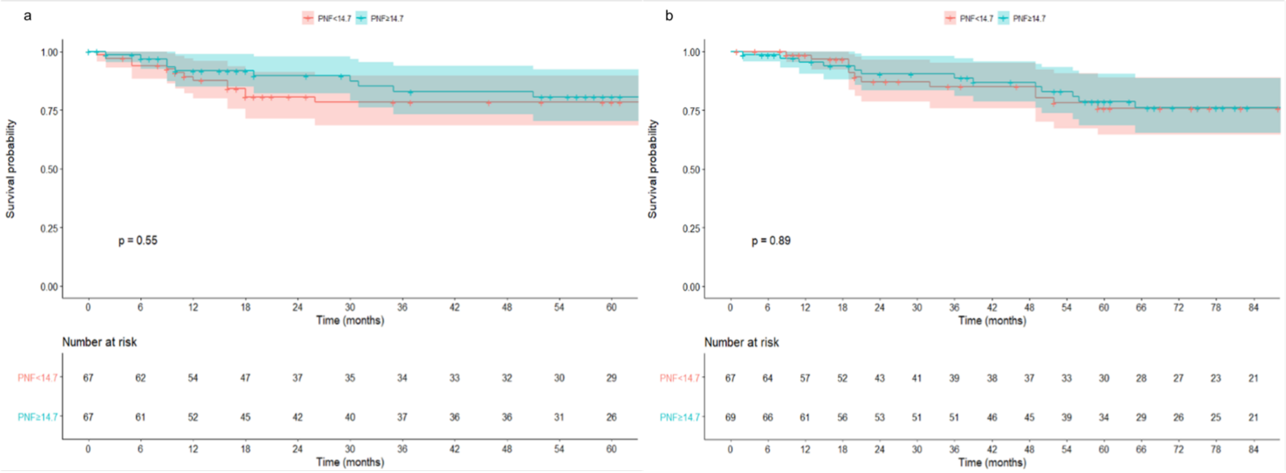

Figure 2. Kaplan-Meier survival estimate for OS and DFS for BMI using a cut-off of 25 (a, c) and 30 (b, d).

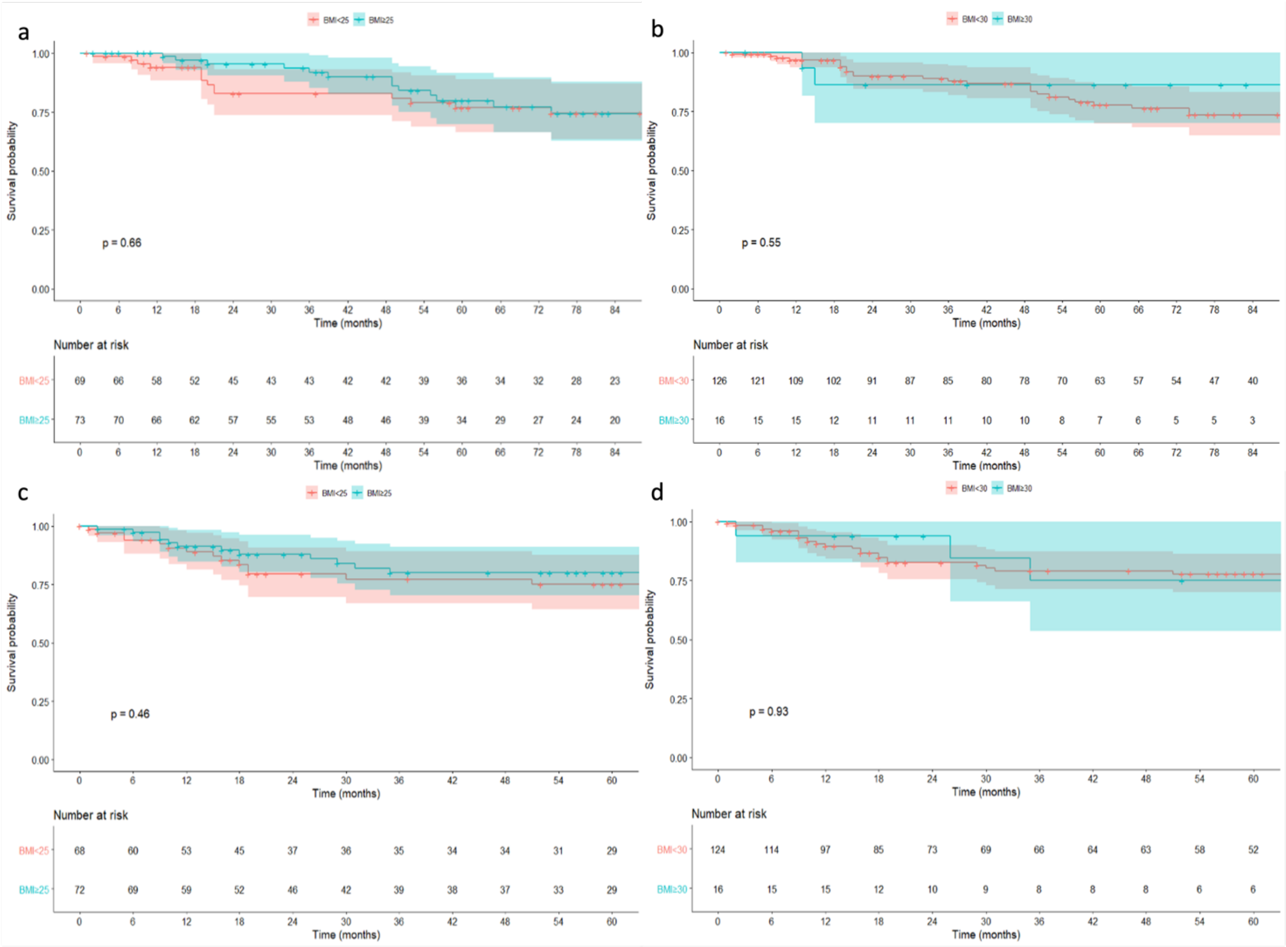

Supplement: Supplementary file 1 [file DataSheet_1.pdf]
